# Modulation of C1-Inhibitor and Plasma Kallikrein Activities by Type IV Collagen

**DOI:** 10.1155/2012/212417

**Published:** 2012-02-12

**Authors:** Sriram Ravindran, Marc Schapira, Philip A. Patston

**Affiliations:** ^1^Department of Oral Medicine and Diagnostic Sciences, College of Dentistry, University of Illinois at Chicago, 801 S. Paulina Street, Chicago, IL 60612, USA; ^2^Department of Oral Biology, College of Dentistry, University of Illinois at Chicago, Chicago, IL 60612, USA; ^3^Department of Hematology, CHUV, University of Lausanne, 1011, Switzerland

## Abstract

The contact system of coagulation can be activated when in contact with biomaterials. As collagen is being tested in novel biomaterials in this study, we have investigated how type IV collagen affects plasma kallikrein and C1-inhibitor. Firstly, we showed C1-inhibitor binds to type IV collagen with a Kd of 0.86 *μ*M. The effects of type IV collagen on plasma kallikrein, factor XIIa, and *β*-factor XIIa activity and on C1-inhibitor function were determined. Factor XIIa rapidly lost activity in the presence of type IV collagen, whereas plasma kallikrein and *β*-factor XIIa were more stable. The rate of inhibition of plasma kallikrein by C1-inhibitor was decreased by type IV collagen in a dose-dependent manner. These studies could be relevant to the properties of biomaterials, which contain collagen, and should be considered in the testing for biocompatibility.

## 1. Introduction

Surface-dependent activation of factor XII and plasma prekallikrein is not believed to be a major component of the normal *in vivo* blood coagulation activation process [1]. However, contact activation readily occurs *in vitro*, as a result of contact with surfaces such as glass, kaolin, and other materials and during procedures such as cardiopulmonary bypass [2–5]. Under conditions such as these, factor XIIa could convert factor XI to factor XIa and result in unwanted thrombin generation. Therefore, it remains critical to understand the mechanisms of contact activation, as this has important implications for the thrombogenic properties and biocompatibility of many materials. For example, novel biomaterials are being developed for wound healing and drug delivery. Notably, many of these materials contain collagen [6–9]. Although collagens are naturally occurring molecules, if they are present in nonphysiological situations or at elevated concentrations, they might have unwanted properties, such as being thrombogenic [10].

C1-inhibitor is a proteinase inhibitor in the serpin family which is an important physiological inhibitor of plasma kallikrein and factor XIIa [11]. Previously we have shown that C1-inhibitor can bind to type IV collagen and that this can modulate the reaction with the complement proteinase C1s [12]. As C1-inhibitor is the main inhibitor of plasma kallikrein, we considered it to be important to investigate the effect of type IV collagen on the inhibition of plasma kallikrein by C1-inhibitor. In this study we have investigated binding of C1-inhibitor to type IV collagen in more detail and studied the effects of type IV collagen on plasma kallikrein and factor XIIa activities and on the inhibition of plasma kallikrein by C1-inhibitor.

## 2. Materials and Methods

### 2.1. Proteins and Reagents

Human plasma kallikrein, factor XIIa, and C1s were from Enzyme Research Labs (South Bend, IN). *β*-factor XIIa was from Calbiochem (San Diego, CA, USA). Human C1-inhibitor was from the Behringwerke (Marburg, Germany). Inactive C1-inhibitor polymers were removed by chromatography on Phenyl-Sepharose as described previously [13]. Human types I, IV, and V collagen and mouse type IV collagen were from Research Diagnostics (Flanders, NJ, USA). The chromogenic substrates S-2302 (for kallikrein, factor XIIa and *β*-factor XIIa) and Spectrozyme C1-E (for C1s) were from DiaPharma (Westchester, OH) and American Diagnostica, (Greenwich, CT), respectively.

### 2.2. Binding of C1-Inhibitor to Type IV Collagen

Biotinylation of C1-inhibitor was carried out by incubating 0.5 mL of C1-inhibitor in PBS (1 mg/mL) and 0.04 mL of freshly prepared biotinylation reagent (2.2 mg NHS-LC-biotin (Pierce, Rockford, IL) in 0.08 mL water) on ice for 2.5 h. Thereafter, excess reagent was removed using a desalting PD10 column (Pharmacia, Piscataway, NJ, USA), with protein detection at 280 nm. To measure the binding affinity, Immulon 2 Dividastrip 96-well plates were coated with 100 *μ*L mouse type IV collagen or bovine serum albumin at 10 *μ*g/mL in carbonate/bicarbonate buffer at pH 7.5, for 18 hrs at 4°C. Wells were blocked with 1% bovine serum albumin, 0.1% Tween 20, in Tris-buffered saline, followed by washing with 0.1% Tween 20 in Tris-buffered saline. Biotinylated C1-inhibitor was added to the wells at 1.9–125 ng/mL. Binding was measured by subsequent incubation with 125-I streptavidin, washing and counting the individual wells in a gamma counter. Affinity was determined by Scatchard plot. To confirm that binding of C1-inhibitor to the type IV collagen was specific, the biotinylated C1-inhibitor was displaced by use of an increasing concentration of unlabelled C1-inhibitor.

Tryptophan fluorescence was also used to analyze the interaction between C1-inhibitor and type IV collagen, using a Photon Technology International fluorimeter. Excitation was at 280 nm, and emission was measured between 290 nm and 400 nm. Samples contained 1 mg/mL C1-inhibitor, 6 *μ*g/mL type IV collagen, or a mixture of both, all in 3 mL of 50 mM Tris-HCl, pH 7.4, 20 mM NaCl.

### 2.3. Effect of Collagen on Proteinase Activity

Plasma kallikrein, factor XIIa, and *β*-factor XIIa were assayed using S-2302, and C1s was assayed using Spectrozyme C1-E, as described previously [14]. To determine the effect of collagen on activity of the proteinases, kallikrein (30 nM), factor XIIa (0.649 *μ*M), *β*-factor XIIa (0.185 *μ*M), and C1s (0.46 *μ*M) were incubated in 20 mM Tris-HCl, pH 7.4, 150 mM NaCl, 0.1% polyethylene glycol 8000 (with kallikrein, factor XIIa, and *β*-factor XIIa) or 1 mg/mL BSA (with C1s), at 37°C in the absence of collagen, or with collagen at 20, 40, 60, 80, or 100 *μ*g/mL. Aliquots were removed at various times for assay of residual proteinase activity.

### 2.4. Effect of Type IV Collagen on Inhibition of Plasma Kallikrein by C1-Inhibitor

The inhibition of plasma kallikrein by C1-inhibitor was measured under pseudo-first-order conditions using a discontinuous assay as described previously [14]. Kallikrein (30 nM) was incubated with a 10-fold excess of C1-inhibitor in 20 mM Tris-HCl, pH 7.4, 150 mM NaCl, 0.1% polyethylene glycol 8000 at 37°C in the absence of collagen, or with collagen at 20, 40, 60, 80, or 100 *μ*g/mL. Aliquots were removed at various times (from 30 seconds to 150 seconds) for assay of residual proteinase activity with S-2302.

## 3. Results

### 3.1. Binding of C1-Inhibitor to Type IV Collagen

**Figure 1:**
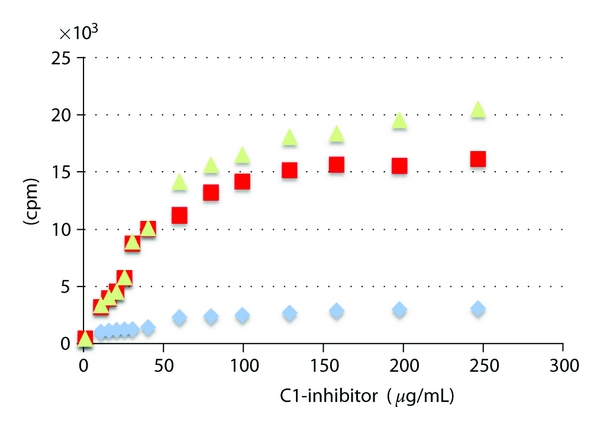
Binding of C1-inhibitor to type IV collagen. The figure shows the binding curve for biotinylated C1-inhibitor to type IV collagen (×), BSA (■), and the collagen binding with the subtraction of the BSA blank (▲). On the *y*-axis is counts per minute (cpm).

**Figure 2:**
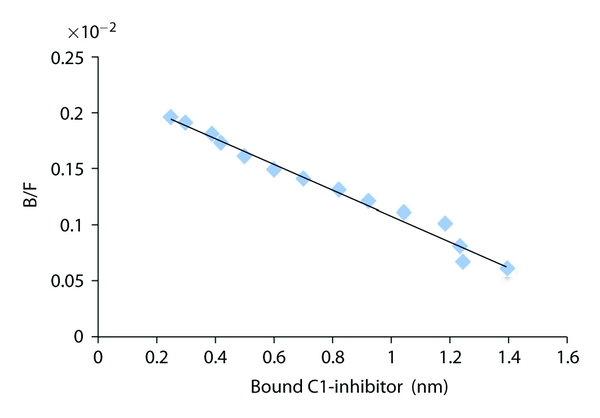
Scatchard plot of C1-inhibitor binding to type IV collagen. Analysis of the data from Figure 1. B/F is the ratio of bound/free biotinylated C1-inhibitor.

**Figure 3:**
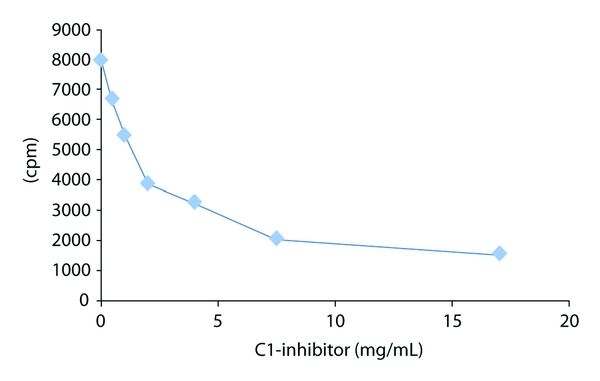
Displacement of biotinylated C1-inhibitor from type IV collagen. To confirm specificity of binding of the biotinylated C1-inhibitor to the immobilized collagen, the labeled protein was displaced with unlabeled C1-inhibitor. On the *y*-axis is counts per minute (cpm).

**Figure 4:**
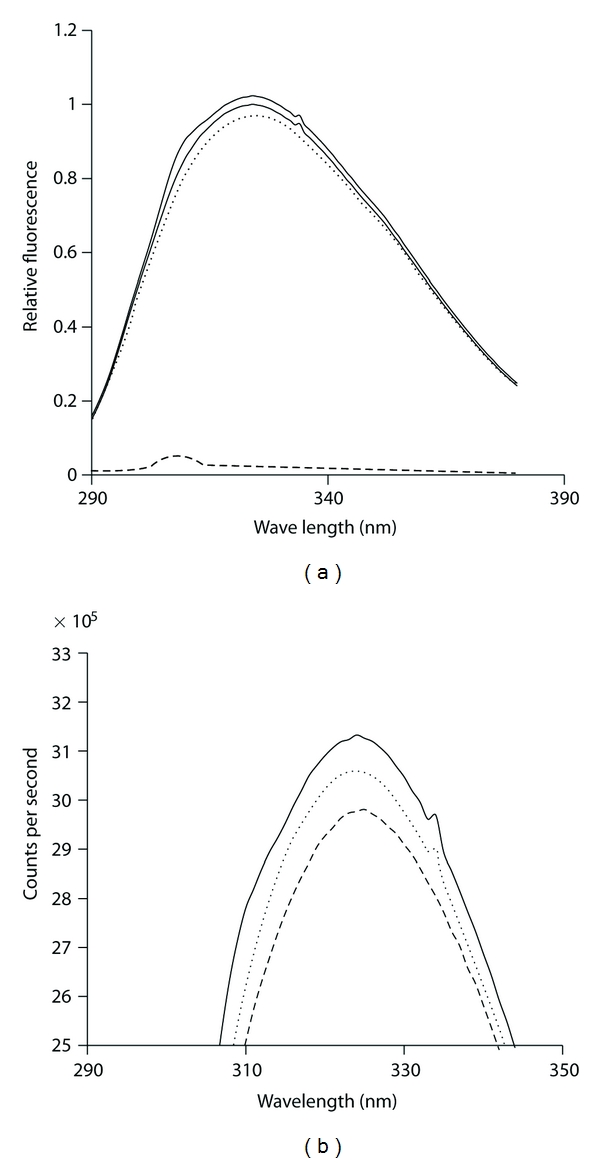
The interaction of type IV collagen and C1-inhibitor assessed by fluorescence spectroscopy. (a) The fluorescence spectra of C1-inhibitor alone (2nd line from top), type IV collagen alone (4th line, at bottom of figure), a mixture of type IV collagen and C1-inhibitor (3rd line from top), and the individual spectra of C1-inhibitor alone and type IV collagen alone added together (1st line at top of figure). (b) An amplified view of the same data at the apex of the peaks (between 295 nm and 355 nm).

We have shown previously in a qualitative assay that C1-inhibitor will bind to type IV collagen [12]. To determine the affinity for this interaction, the binding of biotinylated C1-inhibitor to immobilized type IV collagen was measured (Figure 1). The affinity was determined to be 0.86 *μ*M from the Scatchard plot of this data (Figure 2). Confirmation of the specificity of binding of labeled C1-inhibitor was shown by displacement of the labeled protein by unlabeled protein (Figure 3). Further evidence for the interaction is shown in Figure 4, which shows the fluorescence emission spectra of C1-inhibitor alone, collagen alone, and a mixture of the two. Collagen had very little fluorescence due to the low tryptophan content. The mixture of type IV collagen and C1-inhibitor showed less fluorescence than the C1-inhibitor alone, and less fluorescence than when the individual spectra of type IV collagen and C1-inhibitor were added together. Although no red or blue shift occurred, this small quench is indicative of an interaction between the two proteins. In addition, the magnitude of quench was dependent on the amount of collagen used (not shown). However, as this quench was small, it could not be reliably used to quantify binding affinity.

### 3.2. Effect of Type IV Collagen on Proteinase Activity

**Figure 5:**
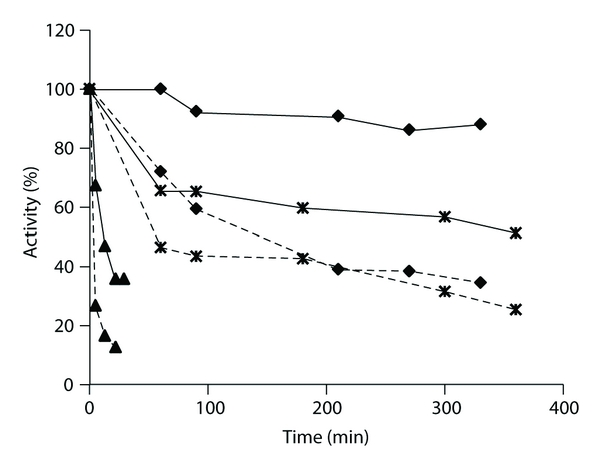
The time course of inactivation of kallikrein, factor XIIa, and *β*-factor XIIa by type IV collagen. Kallikrein was incubated with 20 *μ*g/mL collagen (*◆*) or 100 *μ*g/mL collagen (■), factor XIIa was incubated with 20 *μ*g/mL collagen (▲) or 100 *μ*g/collagen (×), and *β*-factor XIIa was incubated with 20 *μ*g/mL collagen (∗) or 5 *μ*g/mL collagen (•). At the indicated times, a sample was assayed for residual activity with S-2302. The activity is presented as the percentage of the original activity prior to incubation. In the absence of collagen, there was no loss of activity during the same time course.

The effect of type IV collagen on the activity of kallikrein, factor XIIa, or *β*-factor XIIa activity was determined. The proteinases were incubated with increasing amounts of collagen, and the residual activity was measured.  Figure 5 shows a time course of loss of enzyme activity in the presence of 20 or 100 *μ*g/mL of type IV collagen. There was a rapid loss of factor XIIa activity, with 50% of the activity lost after 14.5 minutes in the presence of 20 *μ*g/mL of collagen and 50% of the activity lost after 3 minutes in the presence of 100 *μ*g/mL of collagen. This loss of activity is attributed to factor XIIa adsorbing to the collagen [15]. In the absence of collagen, only 10% of the activity was lost after 30 minutes of incubation. With *β*-factor XIIa, the loss of activity was less, consistent with *β*-factor XIIa not containing the surface binding heavy chain. In the case of kallikrein, 20 *μ*g/mL of collagen caused only a slight loss of activity even after 5 hours, whereas with 100 *μ*g/mL of collagen, there was an initial rapid decrease of activity to about 40%. A similar loss of kallikrein activity by adsorption to surfaces has been observed previously [16, 17], and so these results are entirely consistent with published data. As a control, the complement proteinase C1s showed no loss of activity even after 6 hours with 100 *μ*g/mL of type IV collagen indicating that the effects of collagen were specific for the different proteinases (data not shown).

### 3.3. Effect of Type IV Collagen on the Inhibition of Plasma Kallikrein by C1-Inhibitor

The rate constant for the inhibition of kallikrein by C1-inhibitor in the presence of type IV collagen was determined.  Table 1 shows that there was dose-dependent effect on inhibition. As these assays were performed over a short time frame (under 3 minutes), there was no loss of kallikrein activity during this time (compared to the longer times involved for loss of kallikrein activity seen in Figure 5). The second-order rate constant for inhibition decreased by 50% with 100 *μ*g/mL of collagen. In other studies we have shown that kallikrein inhibition by C1-inhibitor is also reduced when the inhibition reaction is carried out in type IV collagen-coated microtiter plates [18].

## 4. Discussion

In this study we provide evidence that C1-inhibitor can bind tightly to type IV collagen (Figures 1, 2, 3, and 4). We also show that type IV collagen caused a concentration-and time-dependent loss of kallikrein and factor XIIa activities (Figure 5). However, by measuring the inhibition of kallikrein by C1-inhibitor at early time points, we show that type IV collagen can dramatically reduce the rate of inhibition. The most likely explanation for these data is that the binding of C1-inhibitor to collagen reduces the concentration of C1-inhibitor available to react with kallikrein and so the rate of inhibition drops. Given that the plasma C1-inhibitor concentration is *∼*2 *μ*M, this is an interaction which potentially could occur *in vivo*. However, whether this has any regulatory significance for the contact system *in vivo *remains to be determined, particularly as high concentrations of collagen were used. Perhaps a more important consideration is that the effects of collagen on the activities of the proteins tested indicate that studies with biomaterials should consider such reactions as part of their screening for biocompatibility, especially as high local concentrations of collagen would likely be present in such materials. Thus we would suggest that all materials that use collagen (especially type IV) should be evaluated not only for their thrombogenic potential, but also for more specific actions on the contact system proteinases and C1-inhibitor, such as have been carried out with other biomaterials [5, 6, 19–21].

## Figures and Tables

**Table 1:** Effect of type IV collagen on the inhibition of plasma kallikrein by C1-inhibitor.

Amount of type IV collagen (*μ*g/mL)	Second-order rate constant for inhibition of kallikrein (M^−1^s^−1^)
0	16580
10	14370
20	13905
30	11650
40	10510
50	8450
